# Self-administered versus clinician-performed BinaxNOW COVID rapid test: a comparison of accuracy

**DOI:** 10.1128/spectrum.02525-23

**Published:** 2024-02-13

**Authors:** Mary Jane E. Vaeth, Minahil Cheema, Sarah Omer, Ishaan Gupta, Kristie J. Sun, Asia Mitchell, Maryam Elhabashy, Maisha Foyez, Aamna Cheema, Binish Javed, Sophia Purekal, Resham Rahat, Henry Michtalik, Charles Locke, Melinda Kantsiper, James D. Campbell, E. Adrianne Hammershaimb, Yukari C. Manabe, Matthew L. Robinson, J. Kristie Johnson, Lucy E. Wilson, Charles W. Callahan, CONQUER COVID Consortium, Zishan K. Siddiqui

**Affiliations:** 1 Baltimore Convention Center Field Hospital, Baltimore, Maryland, USA; 2 University of Maryland School of Medicine, Baltimore, Maryland, USA; 3 Department of Medicine, The Johns Hopkins University School of Medicine, Baltimore, Maryland, USA; 4 Case Western Reserve University School of Medicine, Cleveland, Ohio, USA; 5 University of Maryland Baltimore County, Baltimore, Maryland, USA; 6 University of Maryland College Park, College Park, Maryland, USA; 7 Atal Bihari Vajpayee Institute of Medical Sciences, Dr. Ram Manohar Lohia Hospital, New Delhi, India; 8 Division of Hospital Medicine, The Johns Hopkins Bayview Medical Center, Baltimore, Maryland, USA; 9 Department of Pediatrics, Center for Vaccine Development and Global Health, University of Maryland School of Medicine, Baltimore, Maryland, USA; 10 Department of Pathology, University of Maryland School of Medicine, Baltimore, Maryland, USA; University of Cincinnati, Cincinnati, Ohio, USA

**Keywords:** COVID-19, BinaxNOW COVID-19 rapid antigen tests, polymerase chain reaction (PCR) testing, clinician-performed tests, self-administered COVID-19 tests, accuracy of COVID-19 tests, COVID-19 detection, COVID-19 test kits

## Abstract

We conducted a single-center study at a free community testing site in Baltimore City to assess the accuracy of self-performed rapid antigen tests (RATs) for COVID-19. Self-administered BinaxNOW RATs were compared with clinician-performed RATs and against a reference lab molecular testing as the gold standard. Of the 953 participants, 14.9% were positive for SARS- CoV-2 as determined by RT-PCR. The sensitivity and specificity were similar for both self- and clinician-performed RATs (sensitivity: 83.9% vs 88.2%, *P* = 0.40; specificity: 99.8% vs 99.6%, *P* = 0.6). Subgroup comparisons based on age and race yielded similar results. Notably, 5.2% (95% CI: 1.5% to 9.5%) of positive results were potentially missed due to participant misinterpretation of the self-test card. However, the false-positive rate for RATs was reassuringly comparable in accuracy to clinician-administered tests. These findings hold significant implications for physicians prescribing treatment based on patient-reported, self-administered positive test results. Our study provides robust evidence supporting the reliability and utility of patient-performed RATs, underscoring their comparable accuracy to clinician-performed RATs, and endorsing their continued use in managing COVID-19. Further studies using other rapid antigen test brands are warranted.

**IMPORTANCE:**

Accurate and accessible COVID-19 testing is crucial for effective disease control and management. A recent single-center study conducted in Baltimore City examined the reliability of self-performed rapid antigen tests (RATs) for COVID-19. The study found that self-administered RATs yielded similar sensitivity and specificity to clinician-performed tests, demonstrating their comparable accuracy. These findings hold significant implications for physicians relying on patient-reported positive test results for treatment decisions. The study provides robust evidence supporting the reliability and utility of patient-performed RATs, endorsing their continued use in managing COVID-19. Furthermore, the study highlights the need for further research using different rapid antigen test brands to enhance generalizability. Ensuring affordable and widespread access to self-tests is crucial, particularly in preparation for future respiratory virus seasons and potential waves of reinfection of SARS-CoV-2 variants such as the Omicron variant.

## INTRODUCTION

The Food and Drug Administration has granted emergency authorization to several COVID-19 rapid antigen tests (RATs), both for clinical use and for patient self-administration at home (self-test). These tests are used for screening asymptomatic individuals as well as diagnosing symptomatic patients (1). Self-test RATs are now widely used for clinical decision-making for the administration of time-sensitive ambulatory therapeutics and infection control (2). The significance and accessibility of self-test RATs were affirmed when the U.S. government distributed 1 billion free tests in January 2022 (3), followed by additional distribution rounds from federal and local governments, fostering widespread testing and aiding in disease control and management.

At-home testing offers several compelling benefits over traditional clinician-administered testing. Widespread distribution in retail pharmacies and free-of-charge tests from public health services led to expedient access. Many individuals also routinely stored these tests at home, significantly lowering traditional barriers to infectious disease testing (4–6). This widespread test availability enabled more timely testing at the onset of symptoms and after disease exposure, potentially earlier than clinician-led alternatives would allow, and thereby promoted more effective early detection strategies.

However, a significant knowledge gap exists in the real-world accuracy of self-test RATs, particularly in the U.S. population (7). Much of the existing evidence base regarding the accuracy of RATs emanates from studies involving clinician-performed RATs (1, 8–11) or from self-testing with reference lab RT-PCR testing (12). The public and clinicians continue to use the results from self-tests, assuming their accuracy is comparable to that of clinician-performed tests. However, self-tests might exhibit a higher rate of false negatives due to inadequate sampling or false positives due to inadvertent sample contamination by untrained individuals or from misinterpretation of visual results. We sought to compare the accuracy of self-testing with clinician-performed RATs.

## MATERIALS AND METHODS

### Study design

In this single-center study approved by the Johns Hopkins Institutional Review Board (IRB), we compared the performance of self-administered BinaxNOW RATs to that of RATs performed by clinicians.

### Participants and study site

After obtaining informed consent, we enrolled adult patients at a walk-up, high-volume COVID community testing site at the State Center from February 12, 2022, to July 15, 2022. This state-owned site, located in an urban setting in Baltimore City, was operated by the Baltimore Convention Center Field Hospital COVID Task Force—a collaboration between the Maryland Department of Health, the University of Maryland Medical Center, and Johns Hopkins Medicine. Test samples were gathered in a semi-permanent, temperature-controlled tent of 11,000 ft^2^, with temperature maintained within the manufacturer-recommended range at all times. English- and Spanish-speaking adults registering at the site received an information sheet about the study (13). They were offered participation when logistical constraints allowed, such as when the site was not overly crowded and adequate staff was available. Everyone visiting the site was offered a free take-home rapid antigen test kit, regardless of study participation. Each study participant was provided with an additional test kit to take home.

### Patient data collection

Before sample collection, demographic data and symptom status were self-reported by the participants. Symptom status was determined based on participants' self-reported responses to the standard Centers for Disease Control and Prevention symptom checklist (14). Participants reporting at least one symptom were classified as “symptomatic,” with the time of symptom onset documented. Those reporting no current symptoms were categorized as “asymptomatic.”

### Sample collection

The collection process began with staff obtaining bilateral anterior nares swabs for direct inoculation onto BinaxNOW rapid antigen clinical tests, followed by bilateral mid-turbinate swabs for RT-PCR testing, a method previously validated (7,15). Participants were then directed to a physically distanced self-test station, where they independently performed the BinaxNOW rapid antigen self-test according to the kit’s instructions. They documented their interpretation of the test results, which were subsequently re-read and recorded by a trained staff member. All study participants provided a clinician-collected RAT sample (clinical test), a clinician-collected PCR sample, then a self-collected and interpreted RAT (self-test). Cycle threshold (CT) count was obtained from the reference lab.

### Statistical analysis

The accuracy of the clinical and self-test RATs (i.e., sensitivity and specificity), along with the corresponding 95% confidence intervals (*CIs*), was calculated using RT-PCR as the gold standard. Self-test results were calculated using participant interpretations of their results.

Correlation coefficient was calculated for self-test and clinical test results. The rapid antigen test results from the self-test and clinician-performed test were compared using the Chi-square test. Subgroup analyses were conducted based on age (≥65 and <65 years), race (African American or White), and symptom status. To evaluate the agreement between participant-reported RAT results and the re-read of these tests by staff, an agreement matrix was constructed, and Cohen’s kappa was calculated using PCR testing as the gold standard. Mean CT counts were compared using the Student’s *t*-test for statistical significance.

## RESULTS

A total of 953 patients participated in the study. Of these participants, 60.6% were female, 58.6% were White, 98.2% were English speaking, the median age was 34.0 years (IQR: 28.0, 48.5), and 34.1% were symptomatic (Table 1). In the study population, 14.9% were positive for SARS-CoV-2 as determined by RT-PCR.

**TABLE 1:** Demographic characteristics of the participants

Characteristics	Participants (*N* = 953)
Female (%)	60.6
Age in years (median, IQR)	34.0 (28.0–48.5)
Hispanic or Latino (%)	5.4
White (%)	58.6
Black or African American (%)	23.2
Asian (%)	7.8
Other (%)	5.1
Baltimore City residence (%)	84.3

The sensitivities of clinician and self-test RATs were similar [88.2%, 95% CI (80.1%, 93.2%) vs 83.9%, 95% CI (75.1%, 90.0%) *P* = 0.40], as were their specificities [99.6%, 95% CI (98.5%, 99.9 %) vs 99.8%, 95% CI (98.8%, 100%) *P* = 0.6]. The correlation coefficient (r) between self-test and clinical test results was 0.91. Sensitivities for self and clinical tests were comparable for different demographic groups, including the elderly (age ≥65 years), younger individuals (age <65 years), African Americans, Whites, as well as symptomatic and asymptomatic groups (Table 2).

**TABLE 2:** Sensitivity of rapid antigen tests by participant characteristics^*a*^

Characteristics	Clinical test *N* (%) [95% CI]	Self-test *N* (%) [95% CI]	*P*-value
All participants	152/177 (85.9%) [79.9–90.7]	146/177 (82.5%) [76.1–87.8]	0.4
Age ≥65 years	8/10 (80.0%) [44.4–97.5]	7/10 (70.0%) [34.8–93.3]	0.6
Age <65 years	144/167 (86.2%) [80.1–91.1]	139/167 (83.2%) [76.7–88.6]	0.4
Race—African American or Black	29/34 (85.3%) [68.9–95.1]	25/34 (73.5%) [55.6–87.1]	0.2
Race—White	92/109 (84.4%) [76.2–90.1]	92/109 (84.4%) [76.2–90.1]	1.0
Symptomatic	135/150 (90.0%) [84.0–94.3]	132/150 (88.0%) [81.7–92.7]	0.6
Asymptomatic	17/27 (63.0%) [42.4–80.6]	14/27 (51.9%) [32.0–71.3]	0.4

^*a*^ PCR test serving as gold standard.

All 177 participants who tested positive for PCR had their self-test RAT cards subsequently re-read by trained staff within 30 min of swab inoculation. Of these, 145 (81.9%; 95% CI, 75.5%–87.3%) were accurately identified by both participants and staff as positive (Concordant True Positive). Nine (5.1%; 95% CI, 2.4%–9.4%) were missed by participants but correctly identified by staff (Participant-Only False Negatives). Furthermore, 22 (12.4%; 95% CI, 8.0%–18.2%) were missed by both groups (Concordant False Negatives). There was a single case (0.5%; 95% CI, 0.01%–3.1%) where the staff incorrectly identified the result as negative, whereas the subject correctly identified it as positive (Staff Read-Only False Negative) (Table 3).

**TABLE 3:** Agreement matrix of self-administered rapid antigen test (self-test) as interpreted by participants and subsequent staff re-read interpretation for PCR-positive test results^*a*^

	Staff interpretation FN	Staff interpretation TP	Total
Self-test FN	22	9	31
Self-test TP	1	145	146
Total	23	154	177

^*a*^ PCR test serving as the gold standard. FN, false negative; TP, true positive.

Participant-Only False Negatives had an average CT count of 27.3 (95% CI, 24.6–30.1), which was significantly higher than that of the Concordant True Positives at 23.7 (95% CI, 22.9–24.4; *P* = 0.01), but lower than that of the Concordant False Negatives at 32.2 (95% CI, 30.4–34.1; *P* = 0.001). The Concordant False Negatives also had a CT count higher than the Concordant True Positives (*P* < 0.0001). Test interpretations of PCR-positive results showed 94.4% interrater agreement between participants and staff, with a Cohen’s kappa of 0.78 (95% CI, 0.65–0.91).

In addition, 776 participants tested negative for PCR, with 773 of these having their self-test cards subsequently re-read by trained staff (Table 4). Test interpretations of PCR-negative results showed 99.4% interrater agreement, with a Cohen’s kappa of 0.44 (95% CI 0.035–0.85).

**TABLE 4:** Agreement matrix of Self-administered rapid antigen test (Self-Test) as interpreted by participants and subsequent staff re-read interpretation for PCR-negative test results^*a*^

	Staff interpretation FP	Staff interpretation TN	Total
Self-test FP	2	3	5
Self-test TN	2	766	768
Total	4	769	773

^*a*^ PCR test serving as the gold standard. FP, false positive; TN, true negative.

## DISCUSSION

Our single-center, community-based, cross-sectional study conducted at an urban state-owned, walk-up, free testing site demonstrated comparable accuracy of participant self-test and clinician-performed COVID-19 RATs (7). Consistent with the World Health Organization’s (WHO’s) minimum sensitivity benchmark for symptomatic individuals, both patient- and clinician-performed RATs exceeded 80% sensitivity in our study cohort. However, we did find that a small percentage (5.2%, 95% CI: 1.53% to 9.48%) of positive results that were read negative as participants could have been missed by participants due to misinterpretation of the self-test card. Staff re-read these tests as true positives. These participants were likely less contagious than those accurately identified as positive by participants, but more contagious than those that were Concordant False Negatives (16). Nonetheless, both testing methods exhibited high specificity across all participant groups. It is important to note that race and ethnicity being considered as factors in the demographic data was driven by the recognition of potential disparities in health literacy, access to healthcare, and sociocultural factors that may influence test interpretation among different racial or ethnic groups.

These findings substantiate the reliability of self-test RATs, bolstering confidence in their use for COVID-19 management by both the public and clinicians (4–6). The comparable accuracy of these self-tests paired with clinician-performed RATs lends confidence to their utility in informing decisions pertaining to isolation and therapeutic protocols (2). Particularly reassuring for physicians prescribing treatment based on patient-reported, positive self-test results is the finding that the RAT false-positive rate is low. Additionally, given that PCR-positive patients, who were missed by self-interpretation but caught by staff re-reads, had higher CT counts than those identified as positive by both, and lower than those missed by both, it is even more critical to perform self-tests immediately at symptom onset or as early as possible within the CDC’s recommended post-exposure testing window (17).

Our study adds a valuable dimension to the existing literature. Previous studies conducted outside of the US have indicated similar performance between self-performed and professionally administered RATs (15, 18–20). However, our study uniquely addresses the real-world accuracy of self-administered RATs within diverse urban settings in the US., filling a significant knowledge gap.

Despite the robust findings, our study carries limitations. The selection process was non-random, and the study site’s workflow and demand influenced subject intake. Self-swabbing proficiency might be artificially amplified due to the learning experience gained from two prior swabs for our sample. However, patients, like our participants who continue to seek clinical tests despite widespread availability of self-tests, may actually possess lower confidence in their self-testing capabilities. Test kits may not always be stored at homes in compliance with manufacturer guidance, as they were at our site (21). Another limitation of our study is the use of CT values from the RT-PCR. This value is impacted by variation in the skill of sample collectors and patient cooperation. Furthermore, CT value thresholds may differ between various commercial kits. However, CT values are widely reported and utilized in studies of RAT accuracy and correlate well with RAT accuracy (8, 9, 22, 23). The participant cohort was confined to English- and Spanish-speaking adults using Abbott BinaxNOW rapid antigen self-test kits exclusively. Consequently, our findings’ applicability to other demographic groups, languages, and RAT brands may be limited. Nevertheless, the strengths of our study are notable. Our real-world setting strengthens the validity of our findings, underpinning their applicability in similar urban settings across the U.S.

Our results also have implications for future research and public health interventions. Future studies should attempt to corroborate these findings across a broader sociodemographic spectrum, in additional settings, and with an array of RAT brands, so as to enhance the generalizability of this study’s results. Understanding factors related to self-test interpretation leading to false-negative readings could help improve the accuracy of self-test RATs.

In conclusion, our study’s findings provide strong evidence of the accuracy of patient-performed RATs, highlighting their equivalent accuracy to clinician-performed RATs. Given the minimal false-positive rate and comparable performance to clinician-performed tests, our findings bolster confidence in the validity of self-test RATs. This allows clinicians to confidently use the results of RAT self-tests in making ambulatory therapeutic decisions, obviating the need for a clinical confirmatory test and potential associated delays (2). Given the expert forecast regarding a possible resurgence of coronavirus variants in the next 2 years, comparable to the severity of the Omicron variant, it is crucial to ensure that self-tests are both affordable and broadly accessible, particularly for future respiratory virus seasons (24).
